# The HIV1 Protein Vpr Acts to Enhance Constitutive DCAF1-Dependent UNG2 Turnover

**DOI:** 10.1371/journal.pone.0030939

**Published:** 2012-01-24

**Authors:** Xiaoyun Wen, Laurieann Casey Klockow, Michael Nekorchuk, Hamayun J. Sharifi, Carlos M. C. de Noronha

**Affiliations:** Center for Immunology and Microbial Disease, Albany Medical College, Albany, New York, United States of America; University Hospital Zurich, Switzerland

## Abstract

**Background:**

The HIV1 protein Vpr assembles with and acts through an ubiquitin ligase complex that includes DDB1 and cullin 4 (CRL4) to cause G2 cell cycle arrest and to promote degradation of both uracil DNA glycosylase 2 (UNG2) and single-strand selective mono-functional uracil DNA glycosylase 1 (SMUG1). DCAF1, an adaptor protein, is required for Vpr-mediated G2 arrest through the ubiquitin ligase complex. In work described here, we used UNG2 as a model substrate to study how Vpr acts through the ubiquitin ligase complex. We examined whether DCAF1 is essential for Vpr-mediated degradation of UNG2 and SMUG1. We further investigated whether Vpr is required for recruiting substrates to the ubiquitin ligase or acts to enhance its function and whether this parallels Vpr-mediated G2 arrest.

**Methodology/Principal Findings:**

We found that DCAF1 plays an important role in Vpr-independent UNG2 and SMUG1 depletion. UNG2 assembled with the ubiquitin ligase complex in the absence of Vpr, but Vpr enhanced this interaction. Further, Vpr-mediated enhancement of UNG2 degradation correlated with low Vpr expression levels. Vpr concentrations exceeding a threshold blocked UNG2 depletion and enhanced its accumulation in the cell nucleus. A similar dose-dependent trend was seen for Vpr-mediated cell cycle arrest.

**Conclusions/Significance:**

This work identifies UNG2 and SMUG1 as novel targets for CRL4^DCAF1^-mediated degradation. It further shows that Vpr enhances rather than enables the interaction between UNG2 and the ubiquitin ligase. Vpr augments CRL4^DCAF1^-mediated UNG2 degradation at low concentrations but antagonizes it at high concentrations, allowing nuclear accumulation of UNG2. Further, the protein that is targeted to cause G2 arrest behaves much like UNG2. Our findings provide the basis for determining whether the CRL4^DCAF1^ complex is alone responsible for cell cycle-dependent UNG2 turnover and will also aid in establishing conditions necessary for the identification of additional targets of Vpr-enhanced degradation.

## Introduction

HIV1 Vpr and its HIV2 and SIV counterparts Vpr and Vpx rely on an ubiquitin ligase, characterized by the components DCAF1, DDB1 and Cullin 4, to execute cellular functions. This complex plays a crucial role in HIV1- and HIV2 Vpr-mediated G2 cell cycle arrest [1], [2], [3], [4], [5], [6], [7]. The same ubiquitin ligase complex is also recruited by the closely related HIV2- and SIV_SM_ Vpx proteins to defeat an antiviral factor that blocks efficient reverse transcription in cells of the monocytic lineage [8], [9], [10], [11]. Each of these functions can be hindered by application of proteasome inhibitors.

HIV1 Vpr is a 96 amino acid protein that is incorporated into virions specifically, in small quantities, through a physical interaction with the p6 portion of the Gag polyprotein [12]. Expression of Vpr in dividing cells can block progression of the cell cycle in the G2 phase after most or all of the cellular chromatin has been replicated [13], [14]. This block establishes intracellular conditions that are similar to those encountered after DNA damage [15]. How HIV benefits from this block remains unresolved, although G2, when chromatin is fully assembled and cells are not dividing, may provide an optimal environment for virus production [16]. Expression of HIV2 and SIV_SM_ Vpr also results in G2 arrest albeit in a lower percentage of cells than in HIV1 Vpr-expressing cultures. The lower level of arrest may be due to lower levels of Vpr expression [17], different interactions with cellular partners or both.

While it is clear that HIV1 Vpr and its HIV2 and SIV_SM_ counterparts require ubiquitin ligase for at least some of their functions, including induction of cell cycle arrest, the mechanism by which Vpr targets host proteins for degradation has not been established. Specifically, it has not been determined whether Vpr recruits new targets to the ubiquitin ligase complex or enhances ubiquitin ligase action on substrates that are already being targeted. The only established substrates for Vpr-enhanced degradation in the presence of the CRL4 complex are the uracil DNA glycosylases, UNG2 and SMUG1. These two enzymes act to hydrolyze the N-glycosylic bond between the uracil base and deoxyribose in the context of DNA and thus initiate base excision repair. Proteasomal degradation of both UNG2 and SMUG1 is accelerated in the presence of HIV1 Vpr [18]. Neither of these two proteins has been linked to Vpr-mediated G2 arrest or shown to be the factor that blocks reverse transcription in macrophages. UNG2 has however been shown to interfere with HIV1 infection in the presence of APOBEC3G unless Vpr is present to diminish UNG2 levels [6]. Curiously, other labs showed that UNG2 is required for efficient HIV1 replication and was recruited into virions by integrase or by Vpr [19], [20]. Mansky *et al.* hypothesized that UNG2, recruited into virions by Vpr, could help to protect viral DNA from dUTP misincorporation during reverse transcription in macrophages or from the effects of cytidine deamination [21]. Indeed virus expressing a Vpr mutant that lacks the capacity to recruit UNG2 had a four-fold higher G-to-A mutation rate than wild-type virus [21]. More recently, Yan *et al.* showed that during infections HIV DNA is heavily uracilated in human immune cells [22]. This work further demonstrated that uracilation helps to shield proviruses from the destructive effects of auto-integration. Norman and colleagues showed that UNG2 plays an important role in Vpr-mediated up-regulation of cellular NK cell ligands [23], another recently described Vpr function [24], [25]. Still other work showed that UNG2 has no significant impact on HIV1 replication [26], [27].

UNG2, regardless of its role in HIV infection, is targeted for accelerated proteasomal degradation in the presence of Vpr [18] and therefore serves as a useful model for understanding how Vpr marks proteins for destruction by the CRL4 ubiquitin ligase complex. In work described here we used UNG2 as a model target for Vpr-directed protein degradation. We extended findings from other labs by showing that Vpr-directed UNG2 degradation, like Vpr-mediated G2 arrest, requires DCAF1 in addition to DDB1 and Cul4 [6], [28]. Importantly, we determined that Vpr is not required for basal levels of CRL4^DCAF1^-mediated UNG2 degradation or for the association of UNG2 with this complex but rather Vpr enhances this process. This is the first evidence to support a model in which Vpr augments the normal turnover of CRL4^DCAF1^ substrates. We further found that Vpr-mediated accentuation of UNG2 turnover is blocked when a Vpr expression threshold is exceeded. The interplay between Vpr levels and UNG2 degradation paralleled the levels of Vpr-mediated G2 cell cycle arrest that we observed. The as yet unidentified cellular factor that is ubiquitinated to cause G2 arrest may thus have interactions with Vpr and the ubiquitin ligase complex that are similar to those of UNG2. This mechanistic explanation of Vpr-mediated degradation will aid future work to identify proteins that are more efficiently degraded in the presence of Vpr and could shed new light on how UNG2 is depleted during normal progression of the cell cycle.

## Results

### CRL4^DCAF1^ physically engages and degrades UNG2

Earlier work that identified the CRL4 ubiquitin ligase complex as a functional cellular partner for HIV1 Vpr did not address the role for Vpr in this complex. Specifically, does HIV1 Vpr act as adaptor to expand the specificity of the ubiquitin ligase complex or to enhance the interaction with a protein that is already a target of the complex? To address this question, we selected UNG2, one of only two known targets of Vpr-mediated degradation, as our model substrate. Schröfelbauer *et al.* showed that UNG2 is ubiquitinated and degraded in a Vpr-dependent manner and that this process relies on the CRL4 ubiquitin ligase complex [6]. This work did not however address whether DCAF1 is required for UNG2 degradation as it is for Vpr-mediated G2 cell cycle arrest [6]. To establish UNG2 as a model for Vpr-mediated degradation, we needed to determine whether DCAF1 is required for Vpr-mediated UNG2 degradation as it is for Vpr-mediated G2 cell cycle arrest.

**Figure 1:**
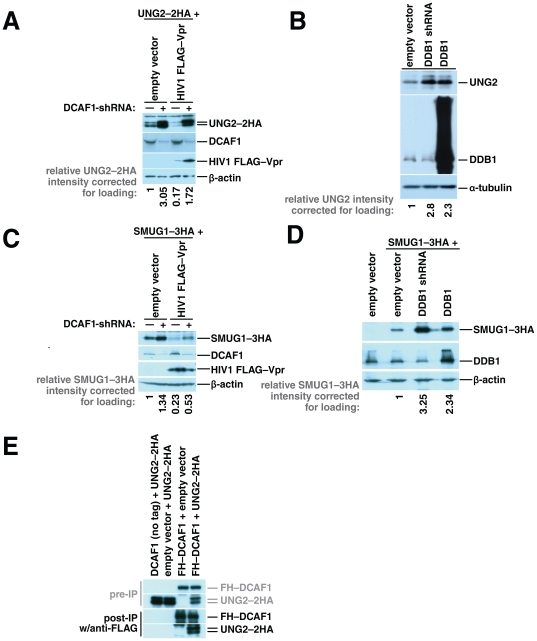
CRL4^DCAF1^ is involved in constitutive turnover of UNG2 and SMUG1. Cultures of 293T HEK cells were transfected with 1 µg of UNG2–2HA expression vector, together with 2 µg of empty vector or 2 µg of an expression vector for DCAF1-directed shRNA and additional empty vector (1 µg) or HIV1 FLAG–Vpr expression vector (1 µg). 48 hours after transfection cell lysates were harvested and tested for expression of UNG2–2HA, HIV1 FLAG–Vpr, endogenous DCAF1 and β-actin by immunoblotting (A). 293T HEK cells were transfected with empty vector (4 µg), or expression vector for DDB1 (4 µg) or DDB1-directed shRNA (4 µg) as indicated. Fourty-eight hours after transfection cell lysates were prepared and tested for expression of endogenous UNG2, DDB1, and α-tubulin by immunoblotting (B). Cultures of 293T HEK cells were transfected with SMUG1-3HA expression vector (1 µg), together with empty vector (2 µg) or an expression vector for DCAF1-directed shRNA (2 µg) and additional empty vector (1 µg) or HIV1 FLAG–Vpr expression vector (1 µg). 48 hours after transfection cell lysates were harvested and tested for expression of SMUG1–3HA, HIV1 FLAG–Vpr, DCAF1 and β-actin by immunoblotting (C). 293T HEK cells were transfected with empty vector (1 µg) or SMUG1–3HA expression vector (1 µg) together with additional empty vector (3 µg) expression vectors for DDB1 (3 µg) or DDB1-directed shRNA (3 µg) as indicated. 48 hours after transfection cell lysates were prepared and tested for expression of SMUG1-3HA, DDB1, and β-actin by immunoblotting (D). 293T HEK cells were transfected with UNG2–2HA expression vector, together with empty vector or an expression vector for untagged DCAF1 or transfected with FLAG–HA–DCAF1 expression vector, together with empty vector or an expression vector for UNG2–2HA. After 48 hours, the cells were lysed and the cleared lysates were incubated with anti-FLAG agarose beads. The bound proteins were eluted with FLAG peptide. The eluted proteins and pre-immunoprecipitation samples were characterized by immunoblotting with HA-specific antibody (E).

If DCAF1 is required for Vpr-mediated UNG2 depletion, then removal of DCAF1 should increase UNG2 levels in the presence of Vpr. To test this we measured levels of UNG2 in cultures transfected with an expression vector for UNG2 with two HA epitope tags at the carboxy-terminus (UNG2–2HA) and either DCAF1-directed shRNA or a non-targeting plasmid in the presence or absence of HIV1 Vpr. Surprisingly, we observed that UNG2 levels increased when DCAF1 expression was perturbed both in the presence and in the absence of HIV1 Vpr (Figure 1A).

This result led us to hypothesize that CRL4^DCAF1^ also mediates turnover of UNG2 in the absence of Vpr, albeit at a lower level. To confirm that UNG2 is degraded by CRL4^DCAF1^, we perturbed a second component of the complex, DDB1, either by reducing its expression using a DDB1-specific shRNA or by over-expressing it from a DDB1-encoding plasmid and measured endogenous levels of UNG2 (Figure 1B). We have demonstrated previously that either over-expressing or reducing expression of DDB1 blocks Vpr-mediated cell cycle arrest [1]. In this experiment, we detected higher endogenous UNG2 levels in cells with either reduced DDB1 expression or DDB1 over-expression compared to cells that express wild-type DDB1 levels. This shows for the first time that CRL4^DCAF1^ determines steady state levels of UNG2. Our observation that UNG2 is turned over by CRL4^DCAF1^ is particularly significant because only one other substrate, Merlin, has been identified for DCAF1 in the context of the CRL4 [29]. Further, our findings show that DCAF1 is required for Vpr-mediated enhancement of UNG2 degradation as it is for Vpr-mediated G2 cell cycle arrest.

Schröfelbauer *et al.* also showed that another uracil DNA glycosylase, SMUG1, is depleted in a Vpr-dependent manner [18]. We reasoned that Vpr-mediated enhancement of normal CRL4^DCAF1^ substrates may be a general mechanism and therefore also tested the levels of SMUG1 in the presence or absence of DCAF1 and DDB1. We found that SMUG1 levels like those of UNG2 increase when DCAF1 or DDB1 is depleted both in the presence or absence of Vpr (Figure 1C and D). SMUG1 is thus the third target for this complex and the second to be degraded more efficiently by this complex in the presence of Vpr.

If CRL4^DCAF1^ targets UNG2 for degradation, then these proteins must assemble within the same complex. We immunoprecipitated DCAF1 from cells co-transfected with UNG2–2HA and FLAG–HA–DCAF1 to test whether DCAF1 and UNG2 assemble and can therefore be co-isolated. Using beads coated with FLAG epitope tag-specific antibody, we were able to co-isolate UNG2–2HA from lysates of cells expressing FLAG–HA–DCAF1 but not from those of cells expressing untagged DCAF1, our negative control (Figure 1E). This physical association further supports a model in which UNG2 associates with the ubiquitin ligase complex through DCAF1 like Merlin and the target that is responsible for Vpr-mediated G2 cell cycle arrest.

### HIV1 Vpr increases UNG2 degradation by enhancing the interaction between UNG2 and the ubiquitin ligase complex

**Figure 2:**
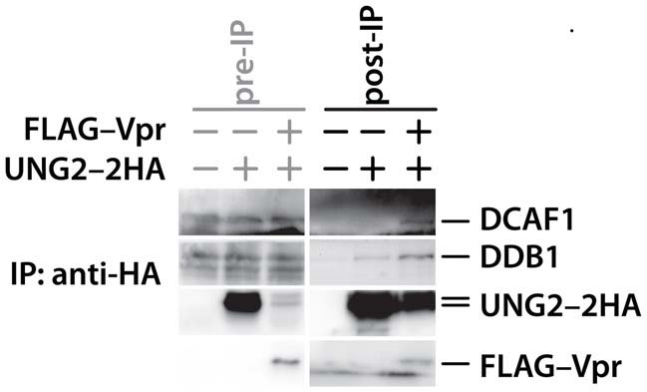
HIV1 Vpr enhances the interaction between UNG2 and the CRL4^DCAF1^ ubiquitin ligase complex. 293T HEK cells were transfected with either UNG2–myc (5 µg, lanes 1 and 4), 5 µg of UNG2–2HA expression vector (lanes 2,3 5 and 6), together with empty vector or expression vector for HIV1 FLAG–Vpr (1.25 µg, lanes 3 and 6). At 48 hours post-transfection, cells were lysed and the lysates were incubated with HA-specific antibody linked to agarose beads. The bound proteins were eluted with HA peptide. The eluted proteins and pre-immunoprecipitation samples were characterized by immunoblotting with antibodies specific for DCAF1, DDB1, the HA epitope tag or the FLAG epitope tag.

Our observation that UNG2 and SMUG1 are targets for the CRL4^DCAF1^ complex in the absence of Vpr compelled us to develop a model in which, rather than acting as an adaptor to recruit new substrates to DCAF1, Vpr enhances the capacity of CRL4^DCAF1^ to target its endogenous substrates. Hrecka *et al.* first raised the possibility that Vpr may be stimulating ubiquitin ligase function through DDA1 [4], but direct interaction between Vpr and DDA1 has not been established. Vpr has however been shown to physically engage both UNG2 and DCAF1 in separate yeast two-hybrid experiments [7], [19]. We therefore reasoned that Vpr could act to enhance the interaction between UNG2 and the ubiquitin ligase complex. If this is the case, then expression of HIV1 Vpr should allow more efficient isolation of DCAF1 with UNG2. To test this possibility we immunoprecipitated HA-tagged UNG2, using HA-specific, antibody-coated beads, from cells transfected with expression vector encoding HIV1 FLAG–Vpr or an empty control vector (Figure 2). Of note, we added Vpr at a level that produces strong UNG2 depletion because that is where we expected Vpr to enhance the interaction between UNG2 and the ubiquitin ligase complex. We therefore treated the cultures with the proteasome inhibitor MG132 for 8 hours prior to harvesting the cell lysates in an effort to increase UNG2 levels in the presence of Vpr. Immunobloting of the isolated proteins revealed that we co-isolated ubiquitin ligase components from both transfection types, however we co-isolated more of these proteins relative to UNG2 in the presence of Vpr. This of course suggests that Vpr enhances the interaction between UNG2 and the ubiquitin ligase complex.

### UNG2 is not degraded when Vpr is expressed at high levels

**Figure 3:**
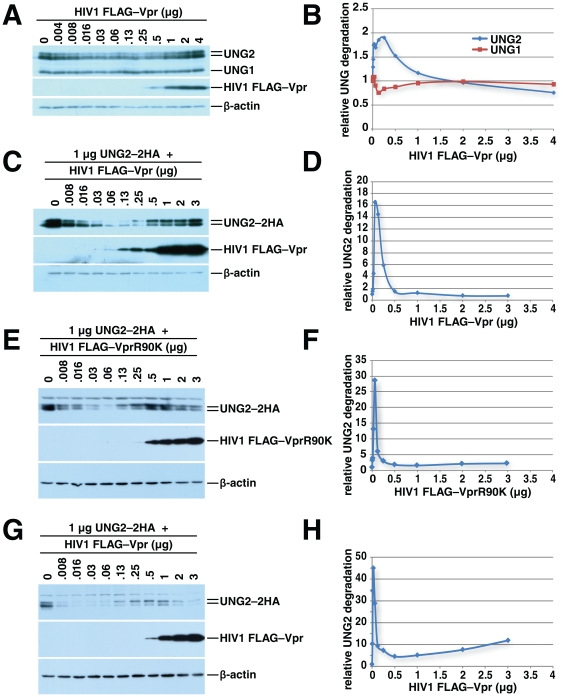
HIV1 Vpr-mediated UNG2 degradation is dose-dependent. 293T HEK cells were transfected with empty vector or increasing amounts of HIV1 FLAG–Vpr expression vector as indicated. 24 hours later the cells were lysed and the expression levels of UNG2, UNG1, HIV1 FLAG–Vpr and β-actin were determined by immunoblotting (A). Quantitation of relative UNG1/2 degradation for Figure 3A was plotted (B). 293T HEK cells were transfected with 1 µg of UNG2–2HA expression vector, together with empty vector or increasing amounts of HIV1 FLAG–Vpr expression vector as indicated. Twenty-four hours later cell lysates were prepared and subjected to immunoblotting with anti-HA, anti-FLAG and anti-β-actin antibodies (C). Quantitation of relative UNG2 degradation for Figure 3C is shown in D. 293T HEK cells were transfected with 1 µg of UNG2–2HA expression vector, together with empty vector or increasing amounts of HIV1 FLAG–VprR90K expression vector as indicated. Forty-eight hours later cell lysates were prepared and subjected to immunoblotting with anti-HA, anti-FLAG and anti-β-actin antibodies (E). Quantitation of relative UNG2 degradation for Figure 3E is shown (F) 293T HEK cells were transfected with 1 µg of UNG2–2HA expression vector, together with empty vector or increasing amounts of HIV1 FLAG–Vpr expression vector as indicated. Forty-eight hours later cell lysates were prepared and subjected to immunoblotting with anti-HA, anti-FLAG and anti-β-actin antibodies (G). Quantitation of relative UNG2 degradation for Figure 3G is shown (H).

UNG2 depletion was incomplete in our initial experiments and appeared to be less pronounced than the levels shown in work by Schroefelbauer *et al*. We reasoned that if Vpr acts as an adaptor between UNG2 and the ubiquitin ligase complex, then adding Vpr should enhance degradation of UNG2. This enhancement should plateau if, for example, Vpr is modifying the binding surface on one partner to allow the second to bind. Vpr could however interfere with recruitment of UNG2 to the ubiquitin ligase complex if high concentrations are able to saturate binding sites on both UNG2 and DCAF1. We thus tested the impact of expressing a range of Vpr levels on UNG2 degradation. The quantities of transfected DNA were held constant with empty expression vector. Western blotting for endogenous UNG2 in whole-cell lysates harvested 24 hours after transfection revealed two phases (Figure 3A and B). The first phase showed an increase in UNG2 depletion with increasing Vpr expression. As Vpr levels increased further, UNG2 depletion decreased. As a control, we measured UNG1 levels. We found these to be relatively constant, but increasing slightly with low levels of Vpr expression. While the effects of Vpr on UNG2 levels appear modest, it is important to note that this experiment examines total levels of endogenous UNG2 in both Vpr-expressing cells as well as non-transfected cells. Significantly, this is the first study to show this bi-phasic pattern of Vpr-mediated degradation with endogenous UNG2.

The two phases of UNG2 expression are more pronounced in cells co-transfected with constant quantities of UNG2 expression vector and increasing quantities of HIV1 Vpr expression vector (Figure 3C and D). This experiment focused on transfected cells, and thus eliminated the background of UNG2 from cells not transfected with Vpr. Importantly, these results confirm that exogenously-expressed UNG2 recapitulates the effects of Vpr on endogenous UNG2 and supports previous studies using exogenous UNG2. The decrease of UNG2 levels, followed by an increase suggests that as Vpr expression surpasses a threshold level, it can no longer promote the interaction between DCAF1 and UNG2. Since Vpr binds to both UNG2, the substrate, and to the ubiquitin ligase complex, through DCAF1, our observation is consistent with a model in which Vpr engages both UNG2 and the ubiquitin ligase complex. At high expression levels Vpr interferes with the assembly of UNG2 with the ubiquitin ligase by saturating binding sites on both UNG2 and DCAF1.

Cellular UNG2 levels have been linked to the cell cycle [30], [31], [32], [33] and could be influenced by overall cell health. We thus tested whether these factors play a role in the dose dependence of Vpr-regulated UNG2 degradation. Schrofelbauer *et al.* demonstrated that VprW54R, which does not assemble efficiently with UNG2 but causes G2 arrest, fails to cause efficient UNG2 depletion [18]. This observation showed that Vpr can accentuate UNG2 depletion beyond levels caused by G2 arrest. We extended these studies by determining whether the concentration-dependent pattern of Vpr-mediated UNG2 depletion could be achieved in the absence of G2 arrest. We tested the HIV1 Vpr mutant, R90K, which has been shown to assemble with UNG2 but yet not trigger G2 arrest [34], [35] or apoptosis [35]. VprR90K like its wild-type counterpart mediated UNG2 depletion at low concentrations, but failed to deplete at higher concentrations (Figure 3E and F). In order to exclude the possibility that Vpr-mediated cell death could be influencing our results, we retained all cells from all of our cultures including any in the media or PBS used for cell washing. Half of each culture was reserved for western blot analysis and the other half for cell viability analysis. Propidium iodide staining of unfixed cells revealed that 48 hours after transfection, all cultures, regardless of whether they were transfected with low or high quantities of VprR90K (Figure 3E and F) or wild-type Vpr (Figure 3G and H), contained equivalent quantities of necrotic cells (Figure S1A and B). The percentage of dead cells varied by no more than two-fold in each sample set while UNG2 was depleted over 25- and 40-fold in the VprR90K and wild-type Vpr samples respectively. We also transfected a separate set of cultures with a wider range of VprR90K and wild-type Vpr concentrations together with a small quantity of GFP expression vector. This allowed us to compare the fraction of necrotic cells in transfected and untransfected cell populations within the same cultures (Figure S1C and D). Again, the differences in necrotic cell fractions could not account for the differences in UNG2 expression. Of note, similar results were seen with a second non-arresting Vpr mutant, VprR80A (Figure S2). This mutant has been reported to act as a dominant negative in G2 arrest assays [5].

### High levels of Vpr expression cause accumulation of phosphorylated UNG2 and redistribution of UNG2 into the cell nucleus

In our Vpr titration experiments we noticed that when Vpr expression reached a level that blocked UNG2 degradation, the doublet representing UNG2 changed from one with a predominant lower molecular weight band to one with a predominant higher molecular weight band (Figure 3A, C, E and G). The upper band of the doublet represents a phosphorylated form of UNG2 that has been described previously [31]. We reconfirmed that the shift seen in our experiments is due to phosphorylation by phosphatase-treating UNG2 that was immunoprecipitated from cells transfected with an expression vector for UNG2–2HA. The higher molecular weight band of the doublet was depleted after phosphatase treatment (Figure S3). Thus high levels of Vpr caused a shift from the unphosphorylated form of UNG2 to one with a higher degree of phosphorylation.

Hagen and colleagues have shown that cyclin-dependent kinases (CDKs) phosphorylate UNG2 [31]. CDKs are found largely in the cell nucleus while UNG2 is found in both the nucleus and the cytoplasm. Our observations thus prompted us to hypothesize that high levels of Vpr expression may change the distribution of UNG2 from the cytosol to the nucleus where it can be phosphorylated.

**Figure 4:**
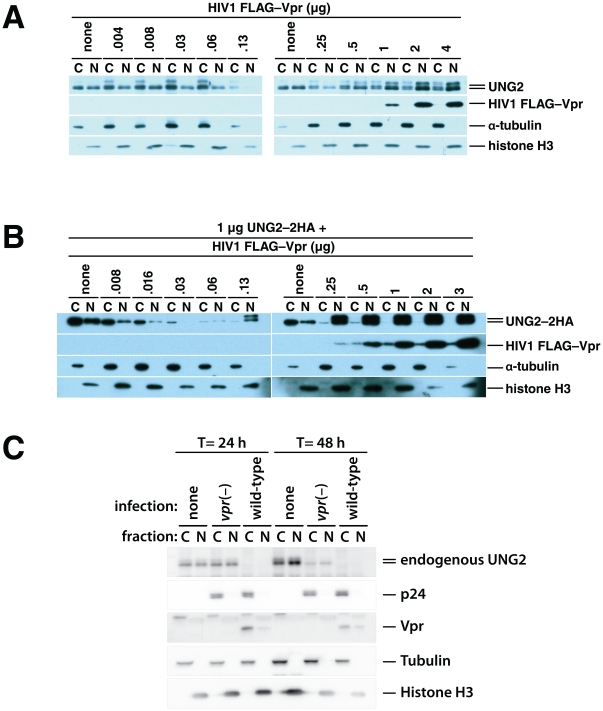
HIV1 Vpr-mediated degradation and subcellular redistribution of UNG2 are dose-dependent. 293T HEK cell cultures were transfected with empty vector alone and together with increasing quantities of HIV1 FLAG–Vpr expression vector as indicated. 48 hours after transfection nuclear and cytoplasmic fractions were prepared and analyzed by immunoblotting with antibodies specific for UNG2, FLAG epitope tag (FLAG–Vpr), α tubulin (cytoplasmic fraction control) and Histone H3 (nuclear fraction control) (A). 293T cultures were transfected with 1 µg of UNG2–HA expression vector alone, together with increasing quantities of HIV1 FLAG–Vpr expression vector as indicated. 48 hours after transfection nuclear and cytoplasmic fractions were prepared. Immunoblotting with anti-HA (UNG2–2HA), anti-FLAG (FLAG–Vpr), anti-α tubulin (cytoplasmic fraction control) and anti-Histone H3 (nuclear fraction control) antibodies was used to determine relative quantities of the respective protein that were present in the fractions (B). HEK 293T cells, either untransfected (left) or transfected with 1 µg of UNG2–2HA expression vector and 3 µg of empty vector (C, right) were mock-infected (none), infected with *vpr(–), env(–)*, VSV-G-pseudotyped virus (*vpr(–)*) or with wild-type, *env(–)*, VSV-G-pseudotyped virus (wild-type).

We repeated the transfections as in the experiments shown in Figure 3, but separated the cells into nuclear and cytosolic fractions (Figures 4A and B). The total UNG2 levels matched those in the whole-cell lysates in Figure 3. As Vpr expression was increased, the levels of endogenous UNG2 decreased both in the cytoplasm and in the nucleus (Figure 4A). However, after Vpr expression was increased further, to the level where UNG2 degradation was blocked, UNG2 accumulated more in the nucleus than in the cytoplasm. Of note, the cytoplasmic and nuclear lanes were loaded with extracts from equivalent numbers of cells. The same patterns were also apparent but more pronounced in cells co-transfected with expression vectors for UNG2–2HA and Vpr (Figure 4B). Cells expressing high levels of Vpr clearly accumulated UNG2–2HA in the nucleus but not in the cytoplasm.

How do UNG2 levels respond to HIV1 infection? In order to relate our titrations to the levels of Vpr expressed by HIV1, we infected HEK 293T cells with vesicular stomatitis G-protein pseudotyped HIV1 and measured UNG2 levels. We used either virus with a deletion in the *vpr* gene (*vpr(–)*) or wild-type virus. Both virus types had a green fluorescent protein gene in place of the *nef* sequence that did not overlap the 3′ U3 region. We infected the cells at a multiplicity of one. At 24 and 48 hours post transfection, we harvested sets of cultures that were uninfected, infected with *vpr(–)* virus or infected with wild-type virus. A portion of these cells were reserved, at 24 hours post infection, for flow cytometry to determine the percentage of infected cells. The remainder of the cells were fractionated to determine the subcellular distribution of endogenous UNG2, p24^gag^ and Vpr. Tubulin and histone H3 were used as fractionation and loading controls.

As early as 24 hours after infection, UNG2 was not detectable in either cell fraction (Figure 4C). The two types of infection were closely matched; both showed equivalent p24^gag^ levels and GFP was detectable in 86% of the *vpr(–)* virus- and 79% of the wild-type virus-infected cells (Figure S4). While the number of infected cells was not identical, the difference in infection could not account for the detected difference in UNG2 levels.

### Attenuation of ubiquitin ligase function promotes UNG2 redistribution that is further enhanced by Vpr

**Figure 5:**
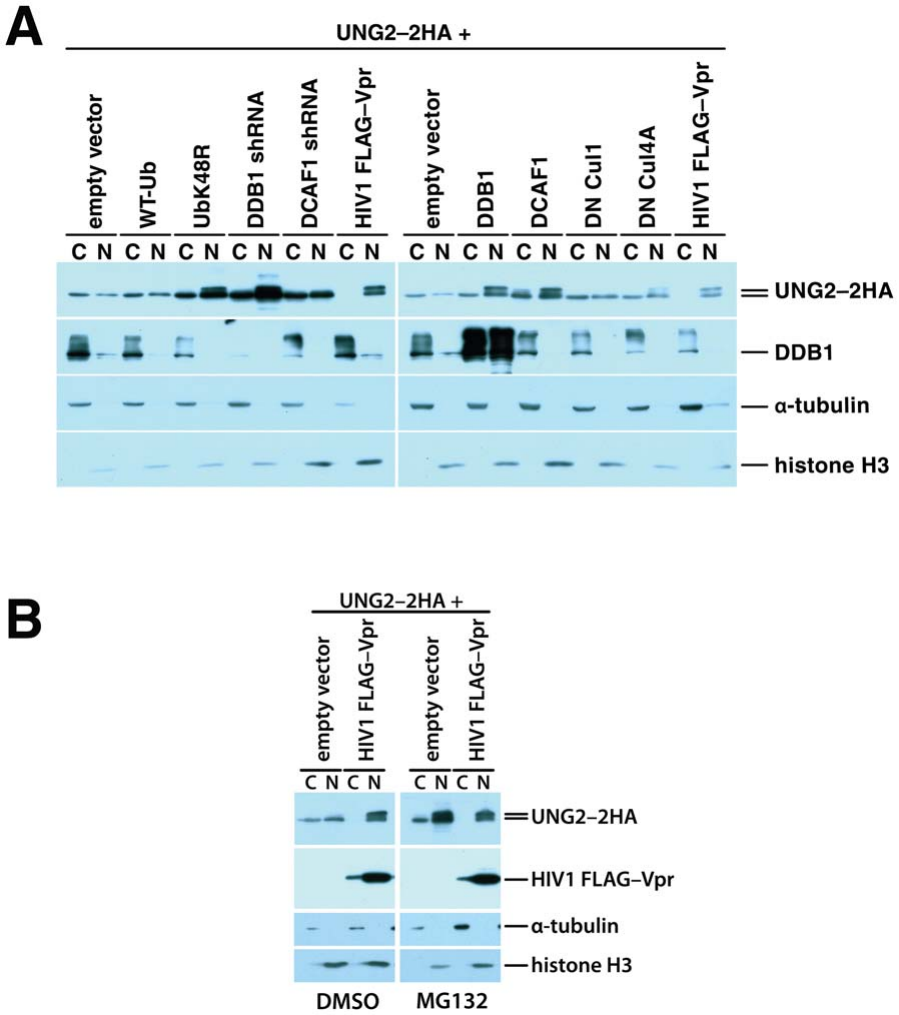
HEK 293T cells were transfected with 1 µg of UNG2–2HA expression vector together with the indicated expression vectors. Forty-eight hours after transfection nuclear and cytoplasmic fractions were prepared. The composition of these fractions was characterized by immunoblotting with HA-, DDB1-,α tubulin- or anti-Histone H3-specific antibodies (A). 293T cells were transfected with UNG2–2HA expression vector, together with empty vector, or expression vector for wild type HIV1 FLAG–Vpr (3 µg). MG132 (12.5 µM) or DMSO (vehicle control) were added 24 hours after transfection. 16 hours later nuclear and cytoplasmic fractions were prepared and characterized by immunoblotting with anti-HA, anti-FLAG, anti-α tubulin (cytoplasmic fraction control) and anti-Histone H3 (nuclear fraction control) antibodies (B).

High levels of Vpr expression failed to target UNG2 for destruction but instead promoted its accumulation in the cell nucleus. We next tested whether nuclear accumulation of UNG2 results from active translocation by Vpr or is a consequence of increased UNG2 stability resulting from decreased ubiquitin ligase action on this protein. To distinguish between these possibilities, we examined whether interfering with UNG2 ubiquitination in the absence of Vpr would affect its subcellular distribution. We perturbed UNG2 degradation by expressing K48R ubiquitin to reduce K48 polyubiquitination, by depleting components of the ubiquitin ligase complex with expression vectors for DDB1- or DCAF1-directed shRNA, by disrupting formation of the complex by over-expressing its components using DDB1 or DCAF1 expression plasmids or by inhibiting the activity of the complex with a dominant negative mutant of Cul4A. We transfected HEK 293T cells with UNG2–2HA expression vector together with empty expression vector and expression constructs for wild-type ubiquitin, K48R ubiquitin, expression vectors for DDB1- or DCAF1-directed shRNA, DDB1, DCAF1, dominant negative Cul1 or dominant negative Cul4A (Figure 5A). As a positive control for UNG2 nuclear translocation we co-transfected another culture with expression vectors for both UNG2–2HA and Vpr.

Overproduction of wild-type ubiquitin and dominant negative Cul1, as expected, did not alter the levels or the distribution of UNG2 detectably (Figure 5A). Expression of DCAF1-directed shRNA increased overall levels of UNG2–2HA but did not change the nuclear to cytoplasmic distribution ratio or phosphorylation status noticeably. Exogenous expression of K48R ubiquitin, DDB1-directed shRNA, DDB1, DCAF1, and dominant negative Cul4A all led to varying increases both in total and nuclear UNG2 levels (Figure 5A). These results show that interfering with UNG2 degradation by expressing K48R ubiquitin or by altering the composition/activity of the CRL4^DCAF1^ ubiquitin ligase complex is sufficient to increase relative levels of UNG2 in the cell nucleus. We observed a similar redistribution of UNG2 into the cell nucleus when we treated UNG2–2HA expressing cells with the proteasome inhibitor MG132 (Figure 5B). Thus, Vpr is not required *per se* for the redistribution of UNG2. However it is important to note that inhibition of the normal UNG2 turnover does not result in complete nuclear translocation like high Vpr expression does (compare the cytosolic fractions of UbK48R, DDB1 shRNA, DDB1, DCAF1, and DN Cul4A to the cytosolic fraction of the Flag–Vpr-transfected samples).

**Figure 6:**
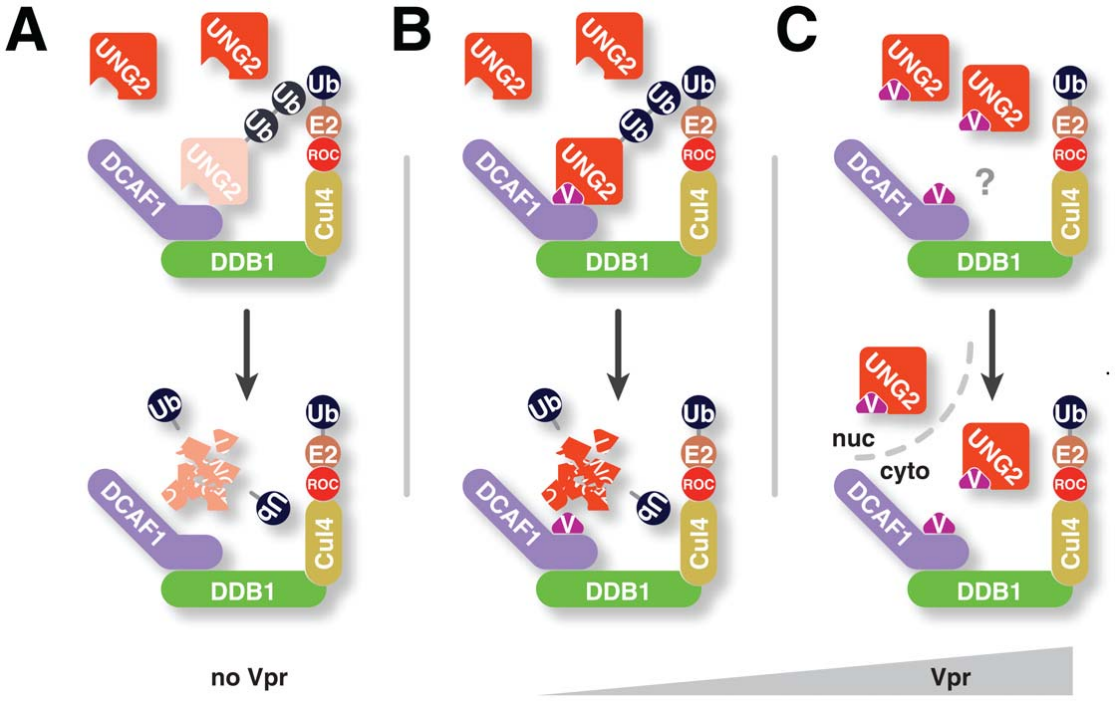
Model for HIV1 Vpr-mediated degradation. In the absence of HIV1 Vpr expression, UNG2 engages DCAF1, is ubiquitinated and thus marked for proteasomal degradation (A). In the presence of HIV1 Vpr, CRL4^DCAF1^-mediated ubiquitination of UNG2 is increased because Vpr enhances the interaction between UNG2 and DCAF1 (B). In the presence of high HIV1 Vpr levels, UNG2 degradation is no longer increased and instead, UNG2 accumulates in the cell nucleus (C).

Nuclear UNG2 accumulation in the presence of high Vpr levels cannot be explained entirely by diminished UNG2 degradation. Importantly these data, showing that either high expression of Vpr or perturbation of the ubiquitin ligase complex causes UNG2 accumulation in the nucleus, indicate that the two functions are linked in a common pathway. This reinforces our finding that high levels of Vpr block constitutive DCAF1-mediated turnover of UNG2. Altogether, our data support a model in which UNG2 is degraded by CRL4^DCAF1^ via its interaction with DCAF1 in the absence of Vpr (Figure 6A). Vpr enhances the degradation by boosting the interaction between DCAF1 and UNG2 (Figure 6B) and can both interfere with degradation and possibly facilitate nuclear import at high levels (Figure 6C).

### The pattern of Vpr-induced cell cycle arrest mirrors that of UNG2 degradation

**Figure 7:**
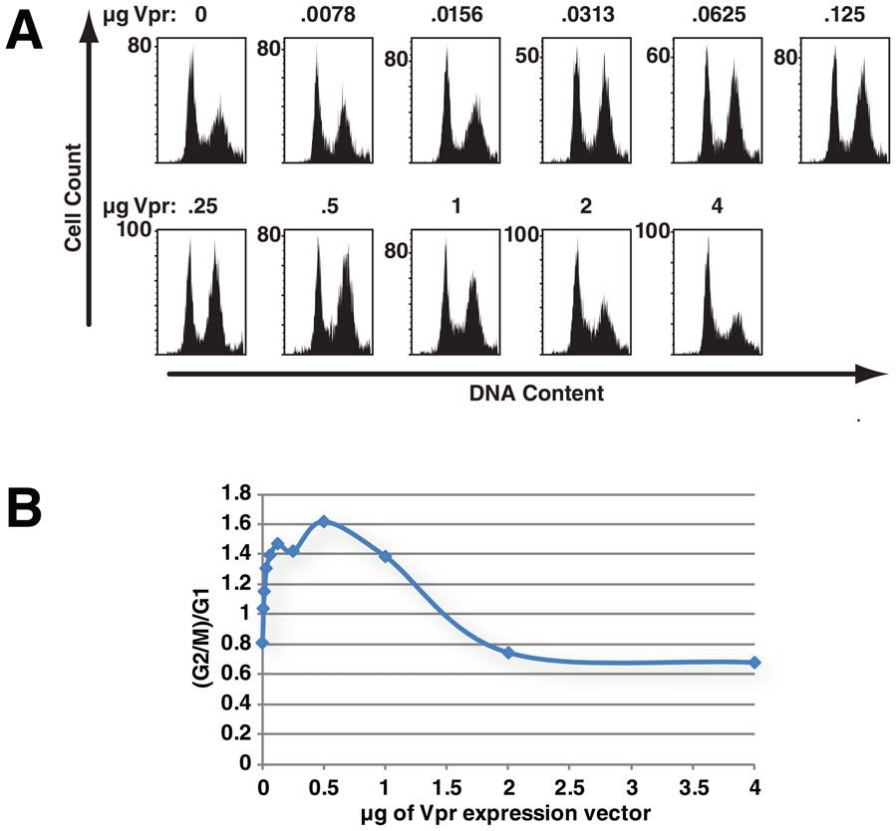
The pattern of Vpr-induced cell cycle arrest mirrors that of Vpr-mediated UNG2 depletion. 293T HEK cells were transfected with empty vector or increasing amounts of HIV1 FLAG–Vpr expression vector as indicated. Total plasmid DNA in each transfection was kept constant by addition of empty vector. The cells in each sample were co-transfected with 175 ng of laminC−GFP to allow identification of nuclei from transfected cells by flow cytometry. The cell nuclei were isolated 48 hours after transfection, treated with RNaseA and stained with propidium iodide. The DNA content was determined by flow cytometry (A). Panel B shows the (G2+M)/G1 ratios for comparison.

Vpr-mediated G2 cell cycle arrest depends on CRL4^DCAF1^ [1], [2], [3], [4], [5], [6], [7], but it is not linked to Vpr-mediated UNG2 degradation [34]. The degradation target responsible for G2 cell cycle arrest has not been identified. We therefore sought to determine whether this unknown target behaves like UNG2 in response to Vpr expression. In devising strategies to identify the cellular protein that is ubiquitinated in response to Vpr expression to trigger arrest, it will be important to determine whether it can be protected by Vpr over-expression. In order to test whether Vpr expression levels influence the efficiency of Vpr-mediated G2 arrest, we transfected HEK 293T cells with 175 ng of laminC–GFP expression vector, to allow identification of transfected cells, and a mix of empty expression vector and increasing quantities of Vpr expression vector. We harvested the cells 48 hours after transfection and measured cellular DNA content using flow cytometry. First, the fraction of cells with DNA content indicative of G2/M (4n) increased with the amount of Vpr expression vector that was transfected (Figure 7A and B). This fraction of cells in G2/M reached a plateau and then decreased as levels of Vpr expression were further increased.

Thus, Vpr levels control the efficiency with which Vpr causes G2 arrest in a pattern that parallels Vpr-mediated UNG2 degradation (compare Figure 7B to Figures 3A and 3C). It is therefore likely that Vpr interacts with the target protein responsible for G2 cell cycle arrest phenotype and the ubiquitin ligase in a manner similar to that with which it interacts with UNG2. Specifically, low levels of Vpr aid ubiquitin ligase function, but high levels interfere with this process.

## Discussion

In work described here, we showed for the first time that DCAF1 and the ubiquitin ligase complex that includes DDB1 and Cul4 play important roles in the turnover of UNG2 and SMUG1 in the absence of Vpr. Depletion of DCAF1 using shRNA increased the levels of both in the presence and absence of Vpr. Further, modifications to the composition of the ubiquitin ligase also increased cellular UNG2 and SMUG1 concentrations (Figure 1). Importantly, we were able to co-immunoprecipitate UNG2 with DCAF1, indicating that both can assemble in the same protein complex.

Recent work by Ahn *et al.* showed that in an *in vitro* assembly of CRL4^DCAF1^ components that had been produced in *E. coli*, UNG2 ubiquitination was enhanced in the presence of Vpr [28]. Interestingly however, lower levels of ubiquitinated UNG2 were also apparent in their samples containing CRL4^DCAF1^ in the absence of Vpr. This supports our results indicating that UNG2 is normally targeted by this complex. The work by Ahn also showed that DCAF1 is important for UNG2 degradation but curiously they saw no depletion of exogenous UNG2 in cells unless they co-transfected exogenous DCAF1. This could, of course, be due to the relative quantity of Vpr that they were producing in those cells.

UNG2 levels have been shown to decline after S-phase to then be restored after mitosis. In our experiments we examined UNG2 levels in asynchronous cell populations; our work however sets the stage for determining whether the CRL4^DCAF1^ ubiquitin ligase complex is responsible for this synchronized turnover. UNG2 and Merlin are the only confirmed substrates for the CRL4 complex. Merlin degradation occurs in response to serum stimulation as cells re-enter the cell cycle at G1 while UNG2 degradation occurs just before mitosis. It remains to be seen whether other cellular proteins act to specifically modify, rather than enable, the targeting of the ubiquitin ligase. For example, do cells express Vpr-like proteins that determine whether or when UNG2, Merlin or other proteins will be targeted for ubiquitination?

Expression of HIV1 Vpr enhances UNG2 degradation [18]. Our observations that UNG2 can assemble with the ubiquitin ligase complex in the absence of Vpr and that UNG2 levels can be increased by depleting DCAF1 show that Vpr, rather than being required for the interaction, enhances its outcome. The augmentation could be in the form of more efficient ubiquitin ligase activity. Our data however, showing that we can co-immunoprecipitate endogenously-expressed components of the ubiquitin ligase complex with UNG2 more efficiently from cultures that co-express Vpr indicate that this protein is likely stabilizing the assembly of UNG2 with the ubiquitin ligase complex (Figure 2). This model is supported by a number of studies that strongly suggest that Vpr can engage both UNG2 and the ubiquitin ligase complex individually. Yeast two-hybrid experiments showed that Vpr and UNG2 [19] or DCAF1 [7] can assemble in the absence of other mammalian proteins. Other work highlighted the Vpr mutant W54R which displays a weakened capacity to engage UNG2 and a UNG2 motif (WXXF) that is required for Vpr binding [34], [36]. Studies where Vpr, DCAF1 and UNG2 were mixed *in vitro* and subjected to analytical size exclusion chromatography showed that when DCAF1 WD40 domain, Vpr residues 1–79 and UNG2 residues 99–313 were mixed, all three eluted in the same fraction whereas the separate components eluted in different volumes [28]. All of these observations support the formation of a tri-molecular UNG2•Vpr•DCAF1 complex, although we cannot rule out the possibility that Vpr engages multiple partners on the same complex.

The changes in UNG2 degradation in response to different Vpr levels are also consistent with a model in which Vpr enhances the interaction between UNG2 and the ubiquitin ligase complex. If Vpr can engage both UNG2 and DCAF1 it is conceivable that Vpr can engage either protein to help facilitate the interaction. After sites on both are saturated, in this scenario, it is conceivable that Vpr interferes with assembly of UNG2 with the ubiquitin ligase complex. It is less likely, if Vpr were enhancing ubiquitin ligase activity that the function would first increase and then decrease. Further, it appears that decreasing the ubiquitin ligase function allows UNG2 to accumulate in the cell nucleus, but not to the same extent as it does in the presence of high concentrations of Vpr. It is not clear how Vpr promotes the additional increase in nuclear UNG2, although it is possible that Vpr lends an additional nuclear import signal(s) to this complex [37], [38].

Vpr is a relatively small protein and the amino acid residues that have been associated with UNG2 binding (W54) and DCAF1 binding (Q65) are close together. It will be interesting to determine exactly how the proteins assemble. Vpr has been shown to multimerize [39], [40] and could therefore act as a multimer to enhance protein degradation.

The parallels that we observed between the patterns in Vpr-mediated UNG2 degradation and cell cycle arrest suggest that the findings presented here will have relevance to other targets of Vpr action. The protein target for Vpr-mediated cell cycle arrest remains to be identified. In designing a strategy to discover this cellular protein partner our results show that we must consider the possibility that it is already a target for the CRL4^DCAF1^ ubiquitin ligase complex and that this association could be broken if Vpr is present at high levels. Further, over-expression of Vpr could cause depletion of the target in one subcellular compartment and accumulation in another. Finally, our observations stress the importance of evaluating Vpr-mediated effects at multiple expression levels.

Together the observations described in this work extend our knowledge about UNG2 turnover and demonstrate that Vpr acts in cells to enhance this process. Importantly, these studies also emphasize that it is crucial to test a range of Vpr concentrations during the investigation of Vpr functions because they may only occur at specific expression levels. It thus sets the stage for further studies to determine in greater detail how UNG2 degradation is regulated and also provides important new information regarding the control of protein degradation by HIV1 Vpr that will aid in the identification of targets that are critical for HIV1 replication and pathogenesis.

## Materials and Methods

### Cell cultures

293T HEK cells were cultured in Dulbecco's modified Eagle's medium (DMEM) (Hyclone, Mediatech Inc) supplemented with 10% fetal bovine serum (FBS), 100 U/ml of penicillin, 100 µg/ml of streptomycin and 2 mM L-glutamine at 37°C with 5% CO_2_.

### Harvesting of cell cultures

In order to assure that we were sampling the entire cell population in our assays we harvested all of the cells in each well. Of note however we found no noticeable increase in non-adherent cells with any of the treatment conditions. This was paralleled by the necrotic cell staining shown in Figures S1 and S2.

### Plasmids and cell transfection

The plasmids pcDNA3.1(–)HIV1 FLAG–huVpr, pcDNA3.1(–) DNCul4A, pSport6 FLAG–DCAF1, pSM2c DDB1-directed shRNA and pSM2c DCAF1-directed shRNA were described previously [1]. In order to improve detection sensitivity for UNG2, the corresponding coding sequence was sub-cloned from pcDNA3.1(+)UNG2–HA (provided by Dr. Ned Landau) into pcDNA3.1(–) with the addition of a second HA tag. The expression vector for dominant-negative cullin 1 (DNCul1) was provided by Dr. Zhen-Qiang Pan. The expression vector for T7-tagged DDB1 plasmid was provided by Dr. Pradip Raychaudhuri. The expression vectors for wild-type ubiquitin and the non-branching K48R mutant were from Dr. Ron Kopito.

Cells were transfected using a standard calcium phosphate protocol. When proteasome inhibitor was used, MG132 (12.5 µM) was added 24 hours after transfection and cells were lysed 16 hours later.

### Immunoprecipitation

To determine whether UNG2 co-immunoprecipitates with DCAF1, cells were lysed in 1.0 ml of cold ELB buffer (50 mm HEPES, pH 7.3, 400 mm NaCl, 0.2% Nonidet P-40, 5 mm EDTA, 0.5 mm dithiothreitol, and Complete™ protease inhibitor mixture (Roche Applied Science, as per instructions)). The lysates were cleared by centrifugation. The supernatant was incubated with anti-FLAG M2 agarose resin (Sigma-Aldrich). The beads were subsequently washed three times in 1 ml of lysis buffer before bound proteins were eluted with 200 µg/ml of FLAG peptide (Sigma-Aldrich). Eluted proteins were analyzed by immunoblot with anti-HA antibody.

To determine whether Vpr enhances the interaction between UNG2 and the CRL4^DCAF1^ ubiquitin ligase complex, the cells were lysed in 1.0 ml of cold ELB buffer and subjected to immunoprecipitation with anti-HA-agarose beads. The beads were subsequently washed three times in 1 ml of lysis buffer before bound proteins were eluted with 200 µg/ml of HA peptide (Sigma-Aldrich). Western blots of the pre- and post-immunoprecipitation samples were probed for DDB1, DCAF1, UNG2–2HA and HIV1 FLAG–Vpr.

### Western blot analysis

Analyses were done following standard western blotting procedures. The primary antibodies used were: anti-FLAG M2 (F1804, Sigma-Aldrich), anti-HA monoclonal 12CA5 (Roche), anti-DDB1 (Clone ZMD.05, Invitrogen), anti-DCAF1 (a gift from Dr. Ling-Jun Zhao), anti-UNG antibody (a gift from Dr. Geir Slupphaug), anti-α-Tubulin monoclonal (N-356, Amersham), anti-Histone 3 polyclonal (06-755, Upstate Biotech), and anti-β-actin monoclonal (A5441, Sigma-Aldrich).

### Subcellular fractionation

Cell fractionation was carried out using a protocol described previously [41]. Briefly, cells were suspended in Buffer B (10 mM HEPES pH 7.9, 1.5 mM MgCl_2_, 10 mM KCl and Complete™ protease inhibitor cocktail (Roche)) and incubated on ice for 10 min. subsequently cells were lysed by adding 10% NP-40 during brief vortex mixing to a final concentration of 0.04% NP-40. Whole cell lysates were transferred onto 1 ml, 1 M sucrose cushions. After centrifugation at 2000×g for 10 min, the layer above the interface with the cushion was collected as the cytoplasmic fraction. Nuclei, pelleted below the sucrose cushion, were washed once with Buffer B and then lysed in Laemmli buffer. The volume of the nuclear fraction was adjusted so that gels could be loaded with nuclear and cytoplasmic extracts from equivalent numbers of cells.

### Infection/Fractionation

Molecular clones of HIV-1 lacking the capacity to encode Env or both Env and Vpr and encoding GFP in place of N-terminal *nef* sequences were transfected into 293T cells together with an expression vector for VSV-G. Cell-free supernatants were harvested from these cells 48 hours after transfection. These supernatants were used to infect 293T cells at an estimated multiplicity of infection of one. Cells were harvested 24 and 48 hours after infection. At 24 hours some of the cells were reserved for flow cytometry to re-confirm equivalent infections by detection of virus-encoded GFP (Figure S4). The remainder of the cells was fractionated as described above. The fractions were then analyzed by western blotting for UNG2, for tubulin as a cytoplasmic fraction loading and fractionation control, for histone H3 as a nuclear fraction loading and fractionation control, for p24^gag^ as another control for equivalent infection, and for Vpr, to reconfirm Vpr expression in the corresponding samples.

### Necrosis assay

Cells were harvested 48 hours after cultures were transfected with pcDNA3.1(–) and/or pcDNA3.1(–) HIV1 FLAG–huVpr, HIV1 FLAG–huVprR90K or pcDNA3.1(–) HIV1 FLAG–huVprR80A in the amounts and combinations indicated in Figures S1 and S2. All of the media and washes were collected to assure that all live and dead cells were retained. Cells were trypsinized, washed and then exposed to propidium iodide in PBS (10 µg/ml) for 20 minutes before the fraction of propidium iodide-stained cells was determined using flow cytometry. Cells used as positive staining controls were fixed in 2% formaldehyde for 20 minutes before staining.

### Cell cycle analysis

293T HEK cells were transfected with empty vector or increasing quantities of HIV1 FLAG–Vpr expression vector. Total plasmid DNA was kept constant by adding empty vector. Each transfection contained 0.17 µg laminC–GFP to identify nuclei of transfected cells. At 48 hours post-transfection, cell nuclei were isolated and treated with RNaseA and propidium iodide as described previously [1]. DNA content was measured by flow cytometry.

## Supporting Information

Figure S1 **The fraction of necrotic cells is not linked to Vpr expression at 48 hours post-transfection.** Cells were harvested forty eight hours after transfection with pcDNA3.1(–) and/or pcDNA3.1(–) HIV1 FLAG–huVprR90K (A) or pcDNA3.1(–) HIV1 FLAG–huVpr (B) in the amounts and combinations indicated. All cells, adherent or floating were included in the analysis. Cultures were trypsinized, washed and then half of the cells were exposed to propidium iodide in PBS (10 µg/ml) for 20 minutes before the fraction of propidium iodide-stained cells was determined using flow cytometry. The other half was analyzed for protein content (Figure 3 E-H). Cells used as positive staining controls were fixed in 2% formaldehyde for 20 minutes before staining. A second set of cultures was transfected with pcDNA3.1(–) and/or pcDNA3.1(–) HIV1 FLAG–huVprR90K (C) or pcDNA3.1(–) HIV1 FLAG–huVpr (D), as indicated, together with 0.175 µg/culture of GFP expression vector to allow comparison between transfected and untransfected cell populations. These cultures were treated and analyzed like those in panels A and B above.
(TIF)

Figure S2 **HIV1 Vpr R80A-mediated UNG2 degradation is dose-dependent and Expression of Vpr R80A does not adversely impact cell viability by 48 hours after transfection.** 293T HEK cells were transfected with empty vector or increasing amounts of HIV1 FLAG–Vpr R80A expression vector as indicated. 48 hours later the cells were lysed and the expression levels of UNG2–2HA, HIV1 FLAG–Vpr and β-actin were determined by immunoblotting (A). Quantitation of relative UNG2–2HA degradation was plotted (B). Cells transfected as indicated, were analyzed for propidium iodide staining as in Figure S1, panels C and D (C).
(TIF)

Figure S3 **Phosphatase treatment eliminates the slow-migrating form of UNG2.** 293T HEK cultures were transfected with expression vectors for UNG2–2HA, the K48R ubiquitin mutant together with empty vector or expression vector for HIV1 FLAG–Vpr. At 48 hours post-transfection, the cells were harvested and UNG2–2HA was immunoprecipitated from the lysates with anti-HA agarose beads. The beads were divided into two samples. One sample was treated with calf intestinal phosphatase and the other was incubated in buffer alone. The bound proteins were eluted with HA peptide and immunoblotted for UNG2–2HA.
(TIF)

Figure S4 **The infection efficiency of** ***vpr(–)*** **virus was similar to that of wild-type virus in the experiment shown in** **Figure 4C** **.** Cells reserved at 24 hours post-infection were fixed with formaldehyde and analyzed for GFP fluorescence as in indicator of infection with the GFP-expressing viruses.
(TIF)

## References

[1] Wen X, Duus KM, Friedrich TD, de Noronha CM (2007). The HIV1 protein Vpr acts to promote G2 cell cycle arrest by engaging a DDB1 and Cullin4A-containing ubiquitin ligase complex using VprBP/DCAF1 as an adaptor.. J Biol Chem.

[2] Belzile JP, Duisit G, Rougeau N, Mercier J, Finzi A (2007). HIV-1 Vpr-Mediated G2 Arrest Involves the DDB1-CUL4A(VPRBP) E3 Ubiquitin Ligase.. PLoS Pathog.

[3] Tan L, Ehrlich E, Yu XF (2007). DDB1 and Cul4A are required for human immunodeficiency virus type 1 Vpr-induced G2 arrest.. J Virol.

[4] Hrecka K, Gierszewska M, Srivastava S, Kozaczkiewicz L, Swanson SK (2007). Lentiviral Vpr usurps Cul4-DDB1[VprBP] E3 ubiquitin ligase to modulate cell cycle.. Proc Natl Acad Sci U S A.

[5] DeHart JL, Zimmerman ES, Ardon O, Monteiro-Filho CM, Arganaraz ER (2007). HIV-1 Vpr activates the G2 checkpoint through manipulation of the ubiquitin proteasome system.. Virol J.

[6] Schrofelbauer B, Hakata Y, Landau NR (2007). HIV-1 Vpr function is mediated by interaction with the damage-specific DNA-binding protein DDB1.. Proc Natl Acad Sci U S A.

[7] Le Rouzic E, Belaidouni N, Estrabaud E, Morel M, Rain JC (2007). HIV1 Vpr arrests the cell cycle by recruiting DCAF1/VprBP, a receptor of the Cul4-DDB1 ubiquitin ligase.. Cell Cycle.

[8] Sharova N, Wu Y, Zhu X, Stranska R, Kaushik R (2008). Primate lentiviral Vpx commandeers DDB1 to counteract a macrophage restriction.. PLoS Pathog.

[9] Srivastava S, Swanson SK, Manel N, Florens L, Washburn MP (2008). Lentiviral Vpx accessory factor targets VprBP/DCAF1 substrate adaptor for cullin 4 E3 ubiquitin ligase to enable macrophage infection.. PLoS Pathog.

[10] Hrecka K, Hao C, Gierszewska M, Swanson SK, Kesik-Brodacka M (2011). Vpx relieves inhibition of HIV-1 infection of macrophages mediated by the SAMHD1 protein.. Nature.

[11] Laguette N, Sobhian B, Casartelli N, Ringeard M, Chable-Bessia C (2011). SAMHD1 is the dendritic- and myeloid-cell-specific HIV-1 restriction factor counteracted by Vpx.. Nature.

[12] Singh SP, Lai D, Cartas M, Serio D, Murali R (2000). Epitope-tagging approach to determine the stoichiometry of the structural and nonstructural proteins in the virus particles: amount of vpr in relation to gag in HIV-1 [In Process Citation].. Virology.

[13] Jowett JB, Planelles V, Poon B, Shah NP, Chen ML (1995). The human immunodeficiency virus type 1 vpr gene arrests infected T cells in the G2+M phase of the cell cycle.. J Virol.

[14] Rogel ME, Wu LI, Emerman M (1995). The human immunodeficiency virus type 1 vpr gene prevents cell proliferation during chronic infection.. J Virol.

[15] Roshal M, Kim B, Zhu Y, Nghiem P, Planelles V (2003). Activation of the ATR-mediated DNA damage response by the HIV-1 viral protein R.. J Biol Chem.

[16] Goh WC, Rogel ME, Kinsey CM, Michael SF, Fultz PN (1998). HIV-1 Vpr increases viral expression by manipulation of the cell cycle: a mechanism for selection of Vpr in vivo.. Nat Med.

[17] Kewalramani VN, Park CS, Gallombardo PA, Emerman M (1996). Protein stability influences human immunodeficiency virus type 2 Vpr virion incorporation and cell cycle effect.. Virology.

[18] Schrofelbauer B, Yu Q, Zeitlin SG, Landau NR (2005). Human immunodeficiency virus type 1 Vpr induces the degradation of the UNG and SMUG uracil-DNA glycosylases.. J Virol.

[19] Bouhamdan M, Benichou S, Rey F, Navarro JM, Agostini I (1996). Human immunodeficiency virus type 1 Vpr protein binds to the uracil DNA glycosylase DNA repair enzyme.. J Virol.

[20] Priet S, Gros N, Navarro JM, Boretto J, Canard B (2005). HIV-1-associated uracil DNA glycosylase activity controls dUTP misincorporation in viral DNA and is essential to the HIV-1 life cycle.. Mol Cell.

[21] Mansky LM, Preveral S, Selig L, Benarous R, Benichou S (2000). The interaction of vpr with uracil DNA glycosylase modulates the human immunodeficiency virus type 1 In vivo mutation rate.. J Virol.

[22] Yan N, O'Day E, Wheeler LA, Engelman A, Lieberman J (2011). HIV DNA is heavily uracilated, which protects it from autointegration.. Proc Natl Acad Sci U S A.

[23] Norman JM, Mashiba M, McNamara LA, Onafuwa-Nuga A, Chiari-Fort E (2011). The antiviral factor APOBEC3G enhances the recognition of HIV-infected primary T cells by natural killer cells.. Nat Immunol.

[24] Richard J, Sindhu S, Pham TN, Belzile JP, Cohen EA (2010). HIV-1 Vpr up-regulates expression of ligands for the activating NKG2D receptor and promotes NK cell-mediated killing.. Blood.

[25] Ward J, Davis Z, DeHart J, Zimmerman E, Bosque A (2009). HIV-1 Vpr triggers natural killer cell-mediated lysis of infected cells through activation of the ATR-mediated DNA damage response.. PLoS Pathog.

[26] Kaiser SM, Emerman M (2006). Uracil DNA glycosylase is dispensable for human immunodeficiency virus type 1 replication and does not contribute to the antiviral effects of the cytidine deaminase Apobec3G.. J Virol.

[27] Mbisa JL, Barr R, Thomas JA, Vandegraaff N, Dorweiler IJ (2007). Human immunodeficiency virus type 1 cDNAs produced in the presence of APOBEC3G exhibit defects in plus-strand DNA transfer and integration.. J Virol.

[28] Ahn J, Vu T, Novince Z, Guerrero-Santoro J, Rapic-Otrin V (2010). HIV-1 Vpr loads uracil DNA glycosylase-2 onto DCAF1, a substrate recognition subunit of a cullin 4A-ring E3 ubiquitin ligase for proteasome-dependent degradation.. J Biol Chem.

[29] Huang J, Chen J (2008). VprBP targets Merlin to the Roc1-Cul4A-DDB1 E3 ligase complex for degradation.. Oncogene.

[30] Fischer JA, Muller-Weeks S, Caradonna S (2004). Proteolytic degradation of the nuclear isoform of uracil-DNA glycosylase occurs during the S phase of the cell cycle.. DNA Repair (Amst).

[31] Hagen L, Kavli B, Sousa MM, Torseth K, Liabakk NB (2008). Cell cycle-specific UNG2 phosphorylations regulate protein turnover, activity and association with RPA.. Embo J.

[32] Muller SJ, Caradonna S (1993). Cell cycle regulation of a human cyclin-like gene encoding uracil-DNA glycosylase.. J Biol Chem.

[33] Nagelhus TA, Slupphaug G, Lindmo T, Krokan HE (1995). Cell cycle regulation and subcellular localization of the major human uracil-DNA glycosylase.. Exp Cell Res.

[34] Selig L, Benichou S, Rogel ME, Wu LI, Vodicka MA (1997). Uracil DNA glycosylase specifically interacts with Vpr of both human immunodeficiency virus type 1 and simian immunodeficiency virus of sooty mangabeys, but binding does not correlate with cell cycle arrest.. J Virol.

[35] Jacquot G, Le Rouzic E, David A, Mazzolini J, Bouchet J (2007). Localization of HIV-1 Vpr to the nuclear envelope: impact on Vpr functions and virus replication in macrophages.. Retrovirology.

[36] BouHamdan M, Xue Y, Baudat Y, Hu B, Sire J (1998). Diversity of HIV-1 Vpr interactions involves usage of the WXXF motif of host cell proteins.. J Biol Chem.

[37] Jenkins Y, McEntee M, Weis K, Greene WC (1998). Characterization of HIV-1 vpr nuclear import: analysis of signals and pathways.. J Cell Biol.

[38] Sherman MP, de Noronha CM, Heusch MI, Greene S, Greene WC (2001). Nucleocytoplasmic shuttling by human immunodeficiency virus type 1 Vpr.. J Virol.

[39] Schuler W, Wecker K, de Rocquigny H, Baudat Y, Sire J (1999). NMR structure of the (52-96) C-terminal domain of the HIV-1 regulatory protein Vpr: molecular insights into its biological functions.. J Mol Biol.

[40] Zhao LJ, Wang L, Mukherjee S, Narayan O (1994). Biochemical mechanism of HIV-1 Vpr function. Oligomerization mediated by the N-terminal domain.. J Biol Chem.

[41] Schreiber E, Matthias P, Muller MM, Schaffner W (1989). Rapid detection of octamer binding proteins with ‘mini-extracts’, prepared from a small number of cells.. Nucleic Acids Res.

