# Supplementary figures and images for: The HIV1 Protein Vpr Acts to Enhance Constitutive DCAF1-Dependent UNG2 Turnover
Source: PLoS One. 2012 Jan 24;7(1):e30939. doi: 10.1371/journal.pone.0030939 (PMC3265533; doi:10.1371/journal.pone.0030939)

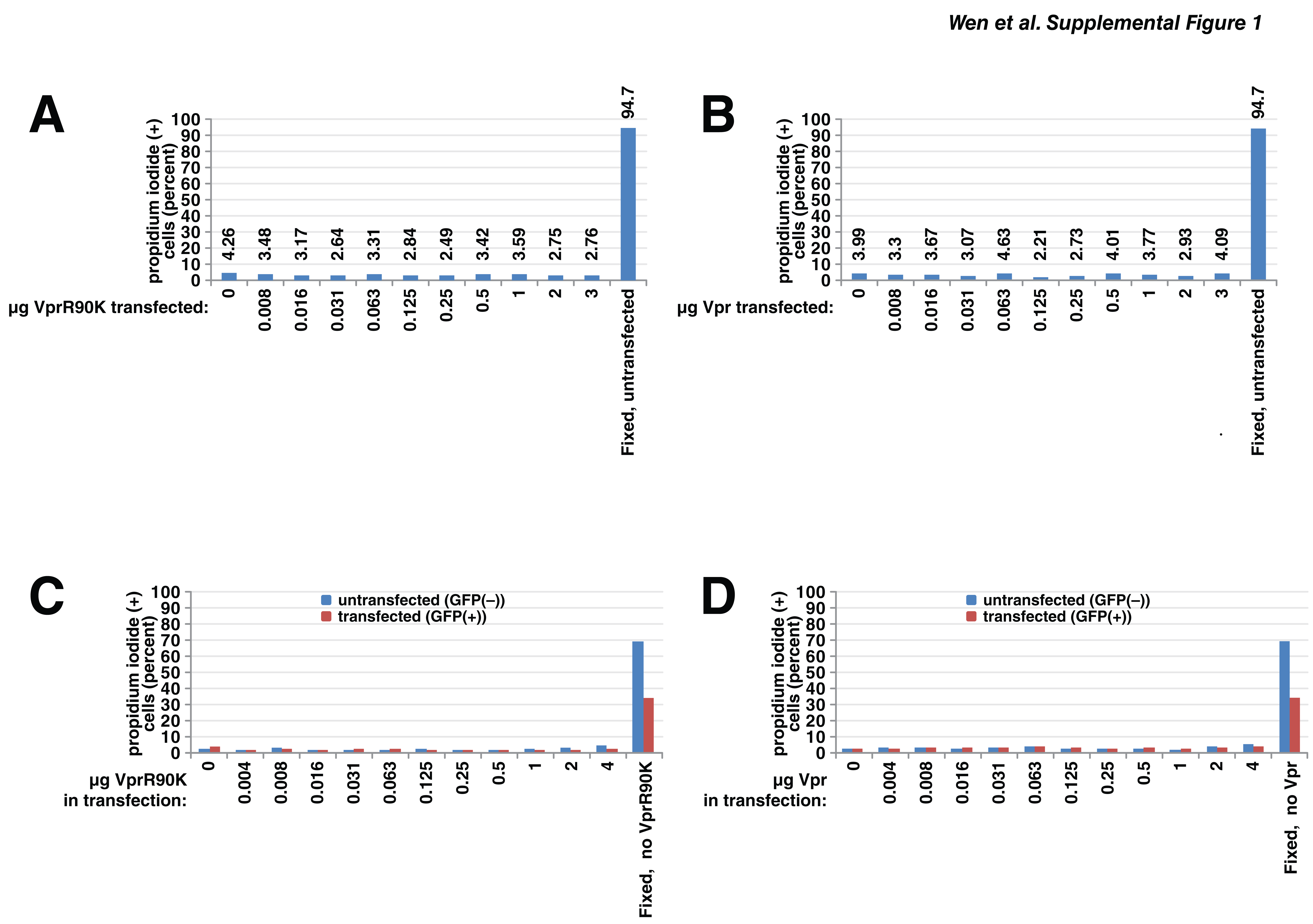

Supplement: Figure S1 — The fraction of necrotic cells is not linked to Vpr expression at 48 hours post-transfection. Cells were harvested forty eight hours after transfection with pcDNA3.1(–) and/or pcDNA3.1(–) HIV1 FLAG–huVprR90K (A) or pcDNA3.1(–) HIV1 FLAG–huVpr (B) in the amounts and combinations indicated. All cells, adherent or floating were included in the analysis. Cultures were trypsinized, washed and then half of the cells were exposed to propidium iodide in PBS (10 µg/ml) for 20 minutes before the fraction of propidium iodide-stained cells was determined using flow cytometry. The other half was analyzed for protein content (Figure 3 E-H). Cells used as positive staining controls were fixed in 2% formaldehyde for 20 minutes before staining. A second set of cultures was transfected with pcDNA3.1(–) and/or pcDNA3.1(–) HIV1 FLAG–huVprR90K (C) or pcDNA3.1(–) HIV1 FLAG–huVpr (D), as indicated, together with 0.175 µg/culture of GFP expression vector to allow comparison between transfected and untransfected cell populations. These cultures were treated and analyzed like those in panels A and B above. (TIF) [file pone.0030939.s001.tif]

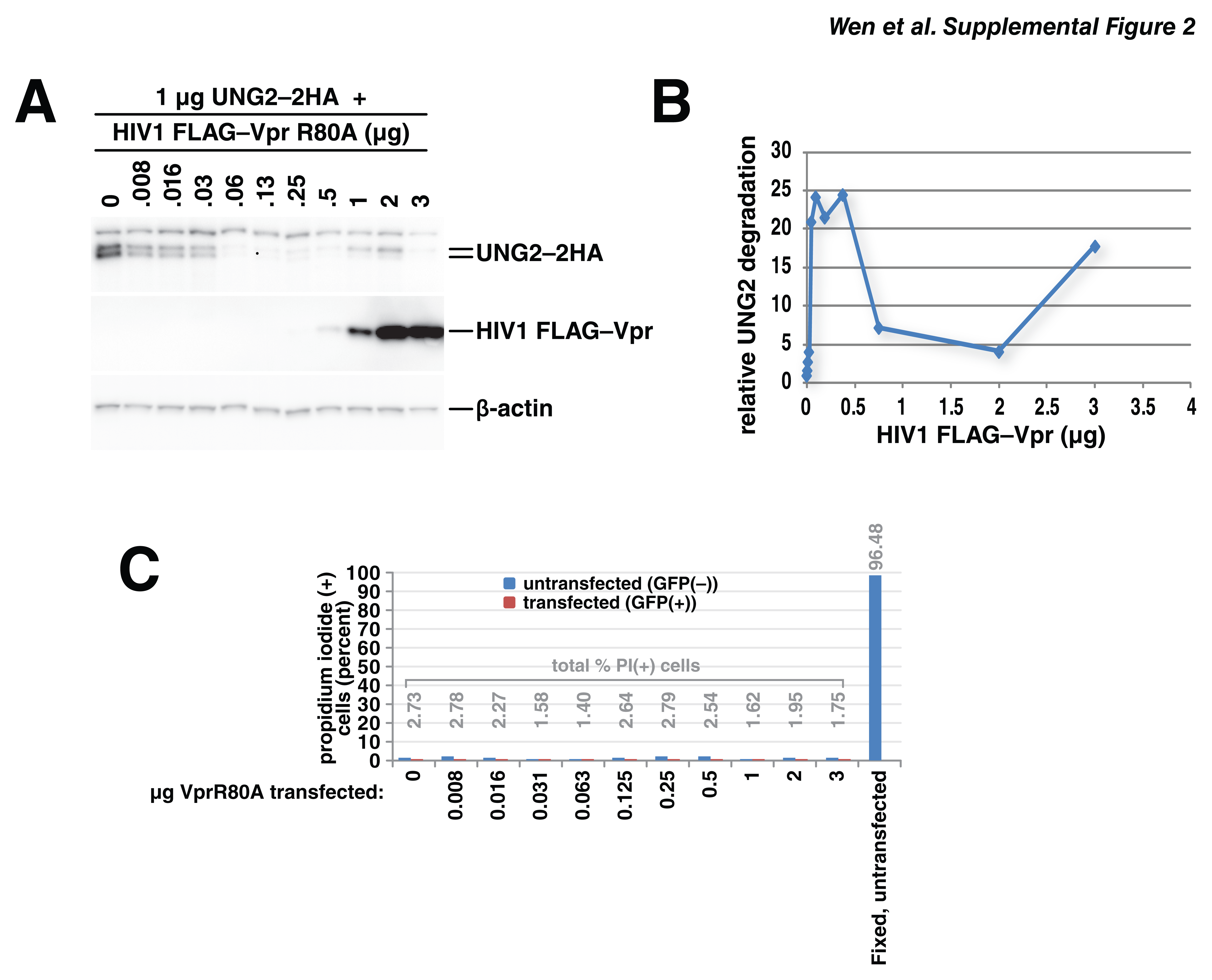

Supplement: Figure S2 — HIV1 Vpr R80A-mediated UNG2 degradation is dose-dependent and Expression of Vpr R80A does not adversely impact cell viability by 48 hours after transfection. 293T HEK cells were transfected with empty vector or increasing amounts of HIV1 FLAG–Vpr R80A expression vector as indicated. 48 hours later the cells were lysed and the expression levels of UNG2–2HA, HIV1 FLAG–Vpr and β-actin were determined by immunoblotting (A). Quantitation of relative UNG2–2HA degradation was plotted (B). Cells transfected as indicated, were analyzed for propidium iodide staining as in Figure S1, panels C and D (C). (TIF) [file pone.0030939.s002.tif]

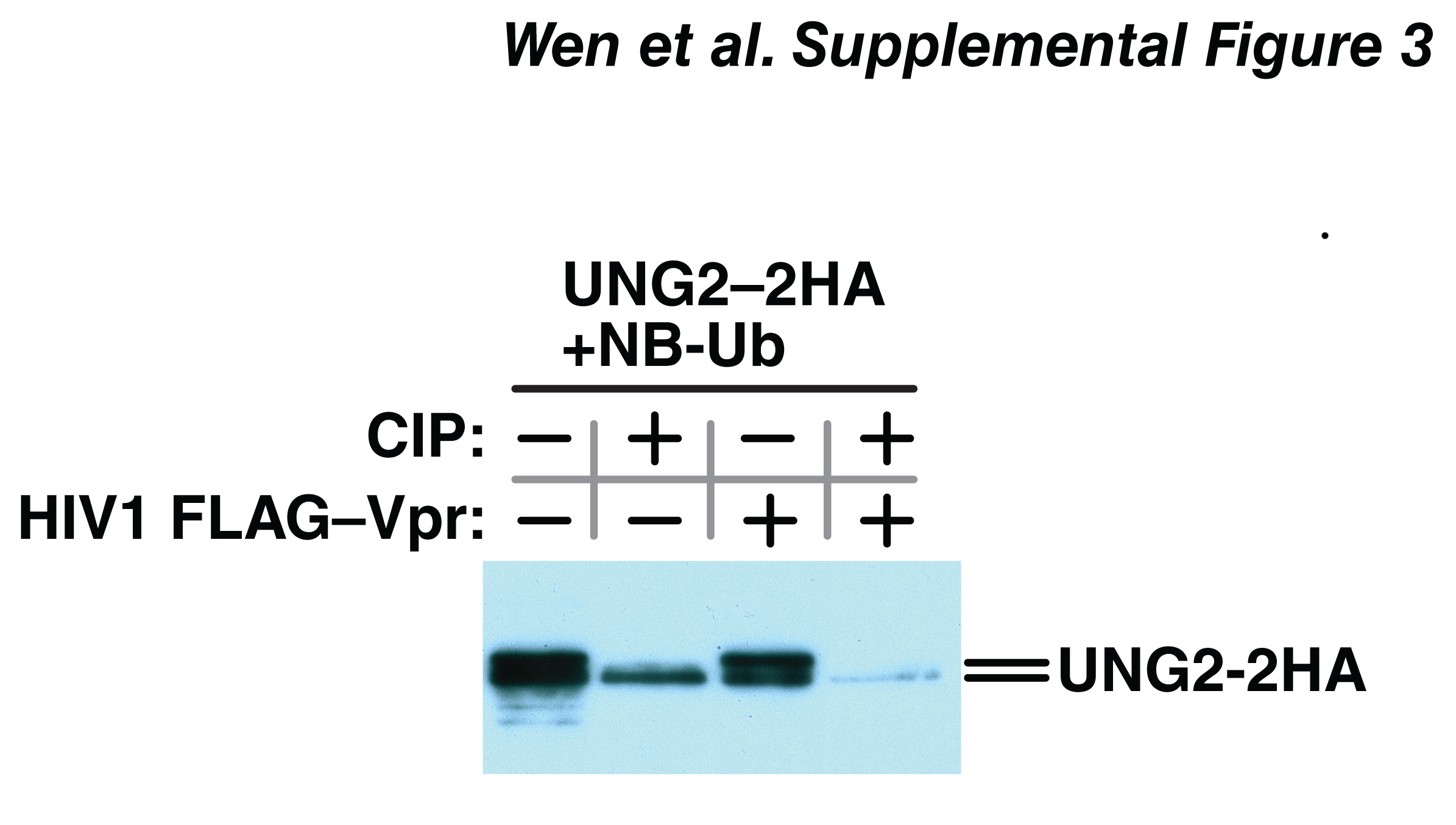

Supplement: Figure S3 — Phosphatase treatment eliminates the slow-migrating form of UNG2. 293T HEK cultures were transfected with expression vectors for UNG2–2HA, the K48R ubiquitin mutant together with empty vector or expression vector for HIV1 FLAG–Vpr. At 48 hours post-transfection, the cells were harvested and UNG2–2HA was immunoprecipitated from the lysates with anti-HA agarose beads. The beads were divided into two samples. One sample was treated with calf intestinal phosphatase and the other was incubated in buffer alone. The bound proteins were eluted with HA peptide and immunoblotted for UNG2–2HA. (TIF) [file pone.0030939.s003.tif]

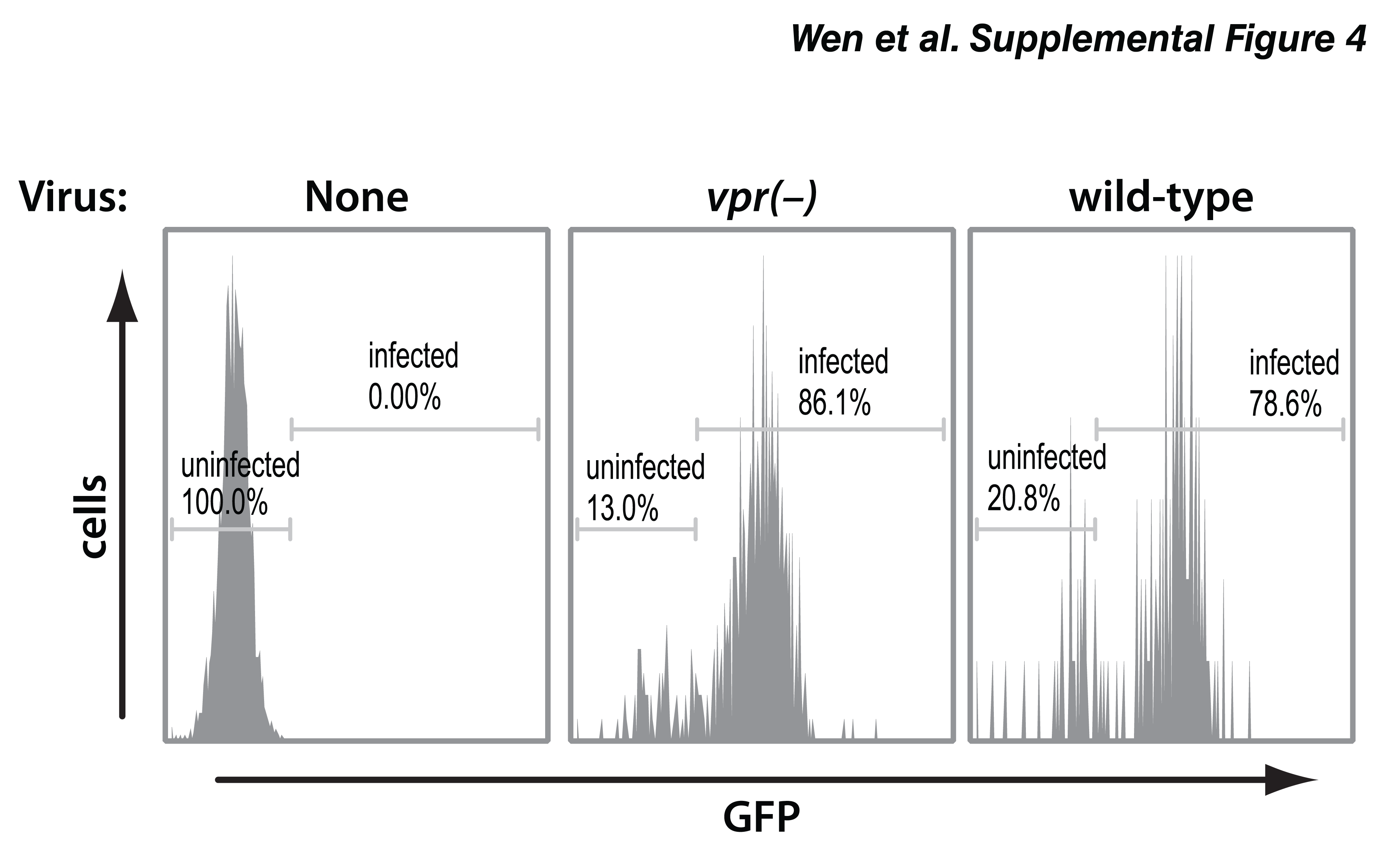

Supplement: Figure S4 — The infection efficiency of vpr(–) virus was similar to that of wild-type virus in the experiment shown in Figure 4C . Cells reserved at 24 hours post-infection were fixed with formaldehyde and analyzed for GFP fluorescence as in indicator of infection with the GFP-expressing viruses. (TIF) [file pone.0030939.s004.tif]
